# Zwitterionic Pathway in the Diels–Alder Reaction: Solvent and Substituent Effects from ωB97XD/6-311G(d) Calculations

**DOI:** 10.3390/molecules30244710

**Published:** 2025-12-09

**Authors:** Agnieszka Łapczuk

**Affiliations:** Cracow University of Technology, Department of Organic Chemistry and Technology Warszawska 24, 31-155 Cracow, Poland; agnieszka.lapczuk@pk.edu.pl

**Keywords:** cycloaddition, norbornene, nitronorbornenes, zwitterionic intermediate, stepwise mechanism

## Abstract

We investigated the Diels-Alder cycloaddition of methylcyclopentadiene with conjugated nitroalkenes and examined the influence of solvent polarity and substituent effects on the reaction mechanism. In nonpolar media (toluene), pathways A and C proceed via a pre-reactive molecular complex (MC), two transition states, and a heterocyclic intermediate, whereas pathways B and D follow a single-transition-state route directly to the norbornene product. Moderate increases in solvent polarity (acetone) do not qualitatively alter the energy profiles or mechanistic patterns, whereas highly polar solvents (methanol, acetonitrile, water, nitromethane) induce a fundamental transformation in pathway B, which adopts a stepwise, zwitterionic mechanism. NPA, MEP, and NCI analyses confirm the polar, charge-separated nature of the zwitterionic intermediate, while BET analysis elucidates the sequential electronic reorganization, highlighting early polarization toward the nitro fragment and stepwise formation of the C-C bonds. Substituent effect studies using Hammett σ parameters reveal that electron-withdrawing groups lower activation barriers, whereas electron-donating groups increase them, indicating that electronic effects dominate over steric factors. Overall, the study demonstrates a general, solvent- and substituent-dependent Diels-Alder mechanism, with pathway B proceeding through a polar, highly asynchronous, stepwise route involving a zwitterionic intermediate.

## 1. Introduction

**Figure 1:**
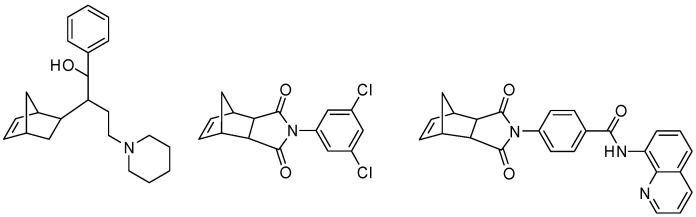
Norbornene-based compounds with applied significance.

The Diels–Alder (DA) reaction is currently one of the most versatile methods for the preparation of the norbornene molecular skeleton [1,2]. This structural motif (Figure 1) represents a key segment of numerous classes of biologically and synthetically relevant compounds, including pharmaceuticals [3,4,5], polymer precursors [6], advanced functional materials [7], and many others.

**Figure 2:**
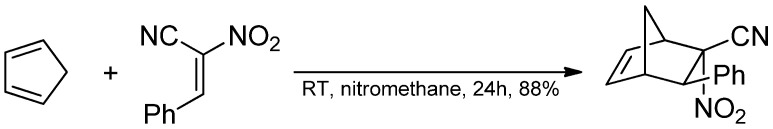
Reaction of cyclopentadiene with *E*-1-cyano-1-nitro-2phenylethene.

This work is a continuation of our previous studies on the mechanistic aspects of the DA reactions [8] between electron-rich conjugated dienes and conjugated nitroalkenes. Previously, we explored DA process with the participation of the cyclopentadiene and E-2-aryl-1-cyano-1-nitroethenes [9]. It was found that the preferred reaction pathway of the starting molecular system consistently leads to the formation of *endo*-nitronorbornene (Figure 2). Interestingly, in reactions conducted in conventional solvents, the preference for the endo isomer increases with the electrophilic character of the substituent [10,11,12], whereas in ionic liquids it increases with the decrease in the electrophilic character of the nitroalkene [13]. This is due to the zwitterionic mechanism of the reaction in the ionic liquid [14]. However, in classical, even very polar solvents, the reaction proceeds according to a one-step mechanism.

**Figure 3:**
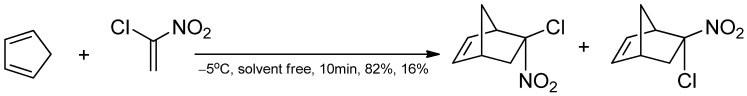
Reaction of cyclopentadiene with 1-chloro-1-nitroethene.

In parallel, we studied DA processes involving the same diene and highly reactive 1-chloro-1-nitroethene [15]. These processes led to a mixture of *endo*- and *exo*-nitronorbornenes (Figure 3), with the former predominating as a stereoisomeric form [16]. The mechanism of these transformations, although asynchronous, is still a single-step process [17].

We have recently analyzed the general trends of changes in the reactivity of nitroalkenes and dienes in DA processes [18], based on the reactivity descriptions estimated within the Molecular Electron Density Theory (MEDT) [19,20,21] framework.

**Figure 4:**
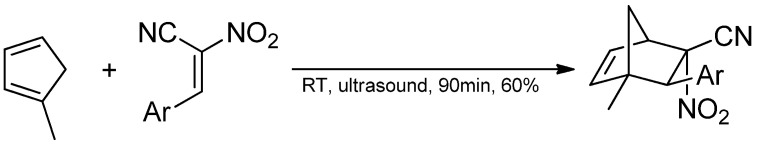
Reaction of 1-methylcyclopentadiene with E-2-aryl-1-cyano-1-nitroethene.

Within the present contribution we decided to perform a full exploration of the molecular mechanism of the DA reaction involving methyl-substituted analog of the cyclopentadiene Cp and homogenous series of E-2-aryl-1-cyano-1-nitroethenes **2a–f**. The mentioned above single step cycloaddition mechanism cannot be however treatment as evident in all cases independently of the nature of addents. In particular, in the case of extremely polar pair of the reaction components, the asynchronicity of the transition state can extended, which can—in an extreme case—lead to the stepwise mechanism [22,23]. This type of mechanism can be realized with the participation of zwitterionic [24] or biradical intermediates [25]. The stepwise mechanism is additionally stimulated by steric effects [26]. This criterium is relevant in the DA reaction between methylcyclopentadiene and E-2-aryl-1-cyano-1-nitroethenes **2a–f** previously analyzed by us on the experimental way (Figure 4) [27].

To address the scientific problem defined above, we aimed to: (i) determine the reaction energy profiles, (ii) focus on the analysis of the zwitterionic structure, (iii) perform a topographical BET analysis of the reaction pathway, and (iv) examine the influence of substituents in the phenyl ring on the reaction course. For this purpose, the results of density functional theory DFT calculations at the ωB97X-D/6-311G(d) level of theory were employed. Similar approach has been recently applied to resolving of mechanistic aspects of many different-type of bimolecular processes including cycloaddidion reactions [28,29].

Earlier calculations [30] performed with the more primitive and now outdated B3LYP functional yielded a different picture of the reaction mechanism. These discrepancies can be attributed to the limitations of the B3LYP method, which does not adequately account for dispersion forces and long-range interactions—effects that are crucial for an accurate description of the studied system. Consequently, the observed divergence not only fails to undermine but actually reinforces the credibility of the results obtained with the more advanced ωB97X-D functional, specifically designed to better capture such subtle electronic and energetic effects [31].

## 2. Result and Discussion

Based on our previous Conceptual Density Functional Theory (CDFT) analyses [18], cyclopentadiene analogues were classified as nucleophilic species and nitroalkenes as electrophiles, indicating that the studied reaction proceeds via a polar mechanism. The Table 1 summarizes the collected data for the studied compounds: methylcyclopentadiene CpMe 1 and nitroalkenes **2a**–**f**, including the Hammett constant σ_p_, global electrophilicity ω, and global nucleophilicity N indices. In the present work, we undertook a more in-depth investigation of the reaction mechanism and offered a broader discussion of its key features.

**Figure 5:**
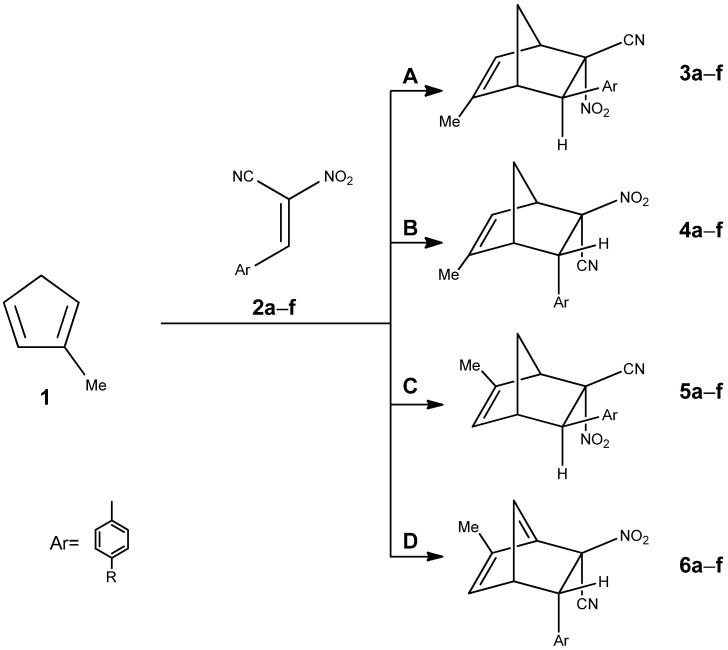
Possible reaction pathways of a cyclopentadiene analog with a conjugated nitroalkene leading to nitronorbornene products.

Previous studies were focused on the analysis of individual substrate properties. Based on these findings, six conjugated nitroalkenes bearing various substituents on the phenyl ring were selected. Both electron-donating and electron-withdrawing substituents were taken into consideration. Among the three CpMe isomers, 2-methylcyclopentadiene was chosen as the representative structure, as it predominates in the isomeric mixture. The conjugated nitroalkenes were classified as strong electrophiles, while 2-methylcyclopentadiene was identified as a strong nucleophile. According to this analysis, carbon C1 in the CpMe molecule was found to be the most nucleophilic site, while carbon C2 in the nitroalkene was identified as the most electrophilic center. Therefore, reaction pathways A and B, involving interactions between these positions, are expected to be the most favorable (Figure 5).

### 2.1. Energetic Profiles and Solvent Effects on the Reaction Mechanism

The first step involved the examination of all four possible reaction pathways. To this end, DFT calculations were performed using the ωB97X-D functional and the 6-311G(d) basis set, in combination with the PCM solvation model.

Calculations were performed for solvents with varying polarity, and it was shown that the reaction course is strongly dependent on solvent polarity. A distinct reactivity pattern was observed in non-polar (e.g., toluene) versus polar solvents (e.g., methanol, nitromethane, acetonitrile, water), with acetone exhibiting intermediate behavior consistent with its polar aprotic nature. Among the solvents investigated, toluene and acetone stand out due to their inability to act as hydrogen bond donors and their relatively low dielectric constants. This sets them apart from more polar, protic solvents such as water or methanol, which are capable of stronger solvation of charged or zwitterionic species. The findings have been collected and are detailed in the subsequent sections of the manuscript.

#### 2.1.1. Stepwise Processes with the Heterocyclic Intermediate

**Figure 6:**
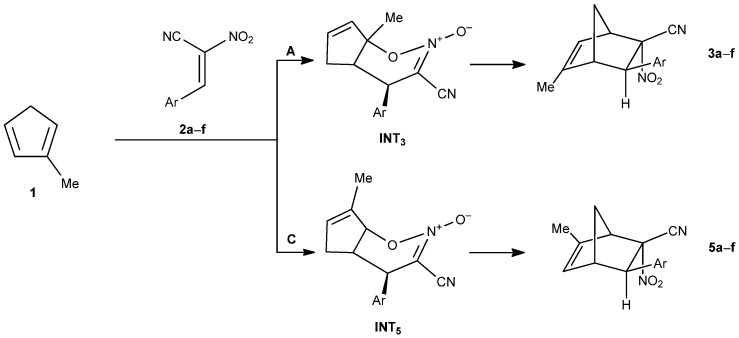
The Diels–Alder reaction between methylcyclopentadiene (**1**) and E-2-aryl-1-cyano-1-nitroethenes (**2a**–**f**) proceeding via pathways A and C.

Regardless of the solvent used, the reaction pathways leading to norbornene derivatives bearing a nitro group in the endo position (pathways A and C, Figure 6) proceed via a two-step mechanism with a heterocyclic intermediate.

The first reaction phase is realized via the formation of a pre-reaction complex (MC), accompanied by a slight increase in Gibbs free energy (ca. 3 kcal/mol). Subsequently, the formation of the transition state (TS1) is associated with a more significant rise in Gibbs free energy, reaching approximately 16–18 kcal/mol (Table 2 and Table 3).

In nitromethane, the reaction proceeds through the formation of a weakly stabilized pre-reactive complex (MC), which is enthalpically favored (ΔH = −8.4 kcal·mol^−1^) but entropically penalized, resulting in a slightly positive free energy (ΔG = +3.1 kcal·mol^−1^). The first transition state (TS1) constitutes a barrier of ΔG^≠^ = 13.4 kcal·mol^−1^ relative to MC (ΔG = +16.5 kcal·mol^−1^ in absolute terms), indicating that the initial bond reorganization is kinetically accessible under ambient conditions. Passage through TS1 leads to the formation of a stabilized intermediate (INT, ΔG = −3.8 kcal·mol^−1^), which lies significantly lower in energy than both MC and TS1.

The subsequent progression toward products requires surmounting a second barrier, with TS2 located at ΔG = +11.4 kcal·mol^−1^. Relative to INT, this corresponds to ΔG^≠^ = 15.2 kcal·mol^−1^, a value higher than the preceding step, suggesting that the second transition state is kinetically more demanding. As a result, the intermediate is expected to be kinetically persistent and may accumulate to measurable concentrations before conversion to the final products. Ultimately, the reaction affords a strongly stabilized product state (PR, ΔG = −10.1 kcal·mol^−1^), confirming that the overall transformation is exergonic.

Taken together, these results demonstrate that in nitromethane the reaction proceeds via a two-step mechanism in which the first barrier (TS1) initiates intermediate formation, while the second barrier (TS2) controls the overall rate.

The calculated thermodynamic and kinetic parameters reveal that the investigated transformation proceeds exothermically in all studied solvents, with reaction enthalpies ranging from −18.7 to −26.7 kcal·mol^−1^ and Gibbs free energies from −2.4 to −10.3 kcal·mol^−1^. The reaction is therefore thermodynamically favorable, although the degree of stabilization of the products varies with the solvent. In particular, acetone appears to diminish the driving force, yielding the least negative ΔG (–2.4 kcal·mol^−1^), whereas toluene and the more polar solvents (methanol, acetonitrile, nitromethane, water) provide a significantly larger stabilization (ΔG ≈ −10 kcal·mol^−1^).

The kinetic profile is dominated by the first transition state (TS1), which consistently constitutes the highest energy barrier across all solvents (ΔG^≠^ ≈ 16–18 kcal·mol^−1^). The second transition state (TS2) is lower in free energy (ΔG^≠^ ≈ 11–16 kcal·mol^−1^), confirming that the first step is rate-determining. Intermediate species (INT) are enthalpically well-stabilized (ΔH ≈ −20 kcal·mol^−1^), but their Gibbs free energies are close to zero or slightly positive in polar media due to the strongly unfavorable entropic contributions (ΔS ≈ −55 cal·mol^−1^·K^−1^). This substantial entropy loss suggests a pronounced ordering effect during complexation and solvation, consistent with the formation of tightly bound intermediates.

Overall, solvent polarity exerts only a minor effect on the kinetic barriers but has a more pronounced influence on the relative thermodynamic stability of the products. These results indicate that the transformation is under kinetic control with a rate-determining first step, while product distribution and thermodynamic stability are modulated by solvent effects.

The reaction pathway in methanol was examined for both mechanistic alternatives (pathway A and pathway C). In both cases, the formation of the pre-reactive complex (MC) is only marginally stabilized (ΔG = +3.1 kcal·mol^−1^ for A and +3.3 kcal·mol^−1^ for C), reflecting a small entropic penalty associated with complexation. The subsequent energy profiles, however, diverge significantly.

For pathway A, the first transition state (TS1) appears at ΔG = +16.5 kcal·mol^−1^, corresponding to a relative barrier of 13.4 kcal·mol^−1^ with respect to MC. This step leads to a stabilized intermediate (INT, ΔG = −3.8 kcal·mol^−1^), from which the second transition state (TS2) requires an additional 15.3 kcal·mol^−1^ to be surmounted. The overall process culminates in an exergonic product state (PR, ΔG = −10.1 kcal·mol^−1^). Thus, pathway A involves moderate and balanced barriers in both steps, making it kinetically feasible and consistent with a mechanism where the intermediate may accumulate before progressing to products.

In contrast, pathway C is associated with higher barriers. TS1 is located at ΔG = +18.7 kcal·mol^−1^, giving a relative barrier of 15.4 kcal·mol^−1^, while TS2 rises to ΔG = +16.4 kcal·mol^−1^, corresponding to a barrier of 20.7 kcal·mol^−1^ above the intermediate (ΔG = −4.3 kcal·mol^−1^). Although the final products are similarly stabilized (ΔG = −9.2 kcal·mol^−1^), the kinetic demands of pathway C are substantially greater.

These results clearly indicate that pathway A is kinetically favored in methanol, with significantly lower activation barriers relative to pathway C. While both mechanisms are thermodynamically viable, the high second barrier in pathway C renders it unlikely to contribute meaningfully under standard conditions, suggesting that the observed reactivity is dominated by pathway A.

#### 2.1.2. Single Step Mechanisms

The energy profile of pathway D in nitromethane reveals a simple scenario without a discrete intermediate. The pre-reactive complex (MC) is only weakly stabilized, with ΔG = +2.9 kcal·mol^−1^, consistent with the entropic cost of association (Table 4). The single transition state (TS) is located at ΔG = +19.6 kcal·mol^−1^, corresponding to a relative activation barrier of 16.7 kcal·mol^−1^ with respect to MC. This makes the process moderately demanding from a kinetic perspective.

**Figure 7:**
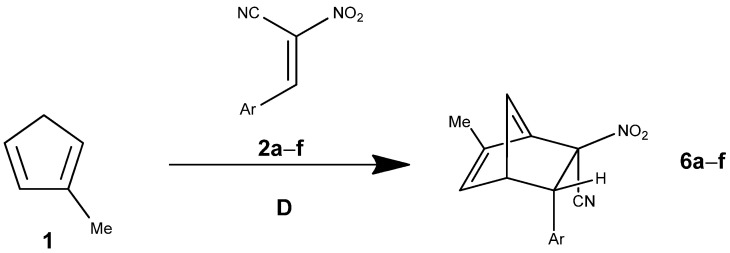
The Diels–Alder reaction between methylcyclopentadiene (**1**) and E-2-aryl-1-cyano-1-nitroethenes (**2a**–**f**) proceeding via pathway D.

The reaction products (PR) are substantially stabilized (ΔG = −8.7 kcal·mol^−1^), confirming that the transformation is exergonic overall. Compared with the more complex mechanisms (pathways A and C), pathway D (Figure 7) is thermodynamically similar (final product stabilization of ca. −9 kcal·mol^−1^) but kinetically less favorable. Specifically, the single-step barrier in pathway D (ΔG^≠^ = 19.4 kcal·mol^−1^) exceeds the initial and secondary barriers of pathway A (16.5 and 15.2 kcal·mol^−1^, respectively) and approaches the higher barriers characteristic of pathway C.

#### 2.1.3. Stepwise Zwitterionic Mechanisms

In contrast to pathways A, C, and D, the reaction proceeding along pathway B exhibits completely different qualitative behavior. In nonpolar solvents (acetone and toluene), the reaction follows a mechanism analogous to pathway D—it is one-step process proceeding directly through the pre-reactive complex (MC) and the transition state to form the final product, nitronorbornene.

However, in more polar solvents, the reaction pathway changes markedly. After the formation of the molecular complex (MC), the system passes through a first transition state (TS1), leading to the formation of a zwitterionic intermediate (ZW). In the subsequent steps, this intermediate evolves through a second transition state (TS2) to yield the final product, nitronorbornene.

This represents one of the few well-documented examples of a reaction proceeding via such a zwitterionic mechanism [32]. Therefore, we focused on a detailed characterization of the zwitterionic intermediate and the corresponding energy profile of this reaction pathway.

**Figure 8:**
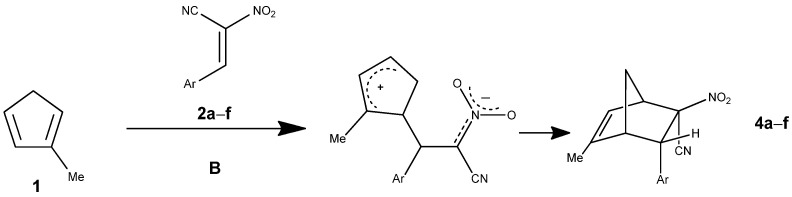
The Diels–Alder reaction between methylcyclopentadiene (**1**) and E-2-aryl-1-cyano-1-nitroethenes (**2a**–**f**) proceeding via pathway B.

In nitromethane, pathway B (Figure 8) proceeds through a sequence involving the pre-reactive complex (MC), a first transition state, a zwitterionic intermediate (ZW), a second transition state, and the final products. The MC is weakly stabilized (ΔG = +3.0 kcal·mol^−1^), while the first transition state lies at ΔG = +18.2 kcal·mol^−1^, giving an activation barrier of 15.0 kcal·mol^−1^ relative to MC (Table 5).

From this transition state, the reaction does not proceed directly to the products but instead forms a zwitterionic intermediate at ΔG = +13.7 kcal·mol^−1^. This species reflects charge separation stabilized by solvation in nitromethane and constitutes a shallow minimum on the potential energy surface. Progression from ZW to the products requires crossing a second transition state (TS2, ΔG = +15.2 kcal·mol^−1^), only slightly higher in energy than ZW itself. The final products are thermodynamically stabilized (ΔG = −9.1 kcal·mol^−1^), confirming a strongly exergonic outcome.

The presence of a zwitterionic intermediate in methanol contrasts with its absence in less polar solvents (toluene, acetone) and indicates that solvent polarity is crucial in stabilizing charge-separated species. In methanol, Pathway B therefore operates as a two-step process with a ZW intermediate, although the overall barrier is comparable to a single-step mechanism.

To gain deeper insight into the polar nature of the reaction, we analyzed the critical structures along the reaction pathway. The evaluation of the global electron density transfer (GEDT) (Table 6) between the interacting fragments allows for a quantitative description of charge flow and provides additional evidence for the polar character of the process.

GEDT values of 0.43 e (TS1) and 0.79 e (TS2) indicate a highly polar process; the markedly larger GEDT for TS2 reflects pronounced charge separation and a stronger zwitterionic character, implying significant electrostatic stabilization and potential solvent sensitivity.

To assess the extent of asynchronicity in the examined reactions, all stationary structures were subjected to detailed analysis. The principal descriptors—interatomic distances between the reactive centers (r), the bond-formation progress parameter (l), and the asymmetry index (Δl)—are compiled in Table 6.

**Figure 9:**
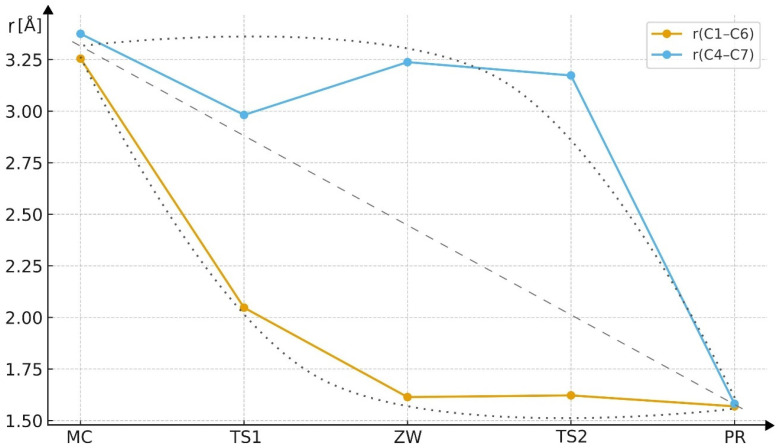
Changes in the C1–C6 and C4–C7 bond lengths along the reaction coordinate (MCB → TS1B → ZWB → TS2B → PRB). The data illustrate an asynchronous, two-step cycloaddition mechanism, where the C1–C6 bond forms prior to C4–C7, leading to a zwitterionic intermediate before complete ring closure.

**Figure 10:**
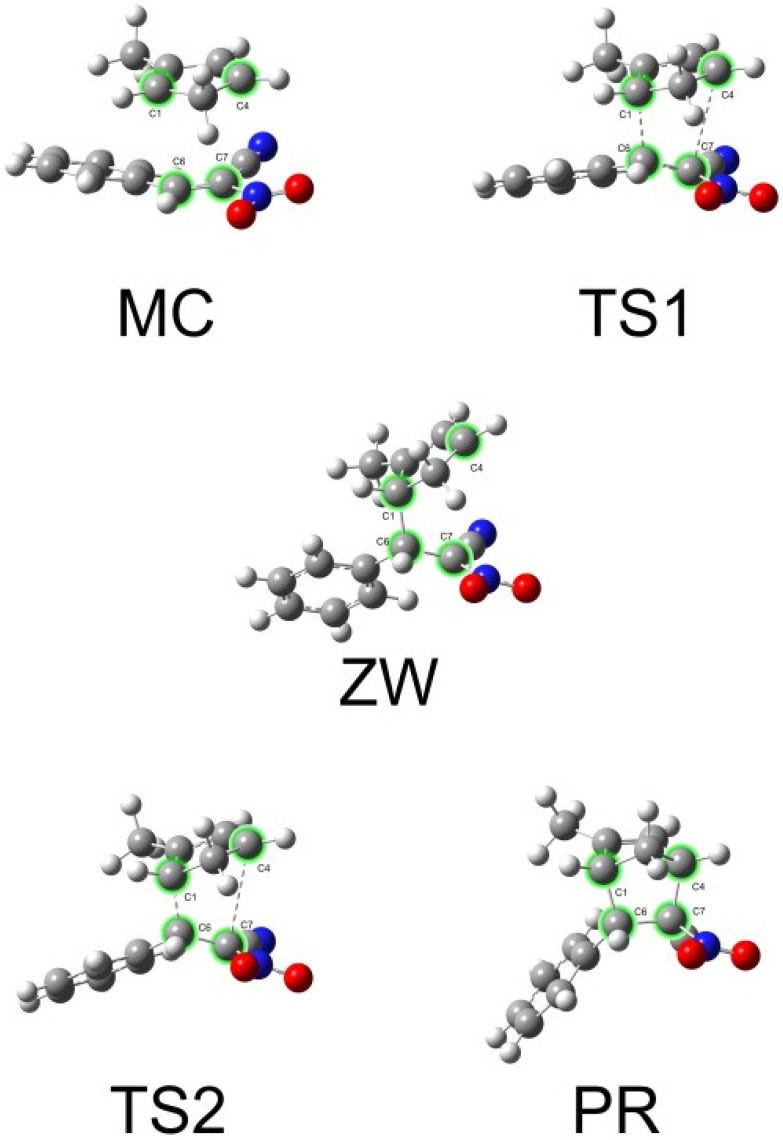
ωB97X-D/6−311g(d): Geometries of the critical structures in the [4 + 2] cycloaddition reaction of 1 and 2c, path B.

Based on the analysis of the C1–C6 and C4–C7 bond lengths along the reaction pathway (Table 6, Figure 9 and Figure 10), it can be clearly concluded that the studied cycloaddition proceeds in an asynchronous, two-step manner. In the initial complex (MC), both distances exceed 3.2 Å, indicating the absence of any significant chemical interaction. In the first transition state (TS1), the C1–C6 distance decreases substantially to 2.05 Å, while C4–C7 remains long (2.98 Å), suggesting that the first bond starts to form earlier. In the zwitterionic intermediate (ZW), the C1–C6 bond is already fully formed (1.61 Å), whereas C4–C7 is still elongated (3.24 Å), supporting the presence of a stepwise mechanism involving a charge-separated intermediate. In the second transition state (TS2), the C4–C7 distance begins to contract, ultimately leading to the formation of the final product, where both bond lengths (≈1.57–1.58 Å) correspond to typical σ bonds within the newly formed ring. These results clearly demonstrate that the cycloaddition does not proceed via a single, concerted process, but rather through two distinct stages characterized by sequential C–C bond formation. The plot shows (Figure 9) the variation of key interatomic distances along the reaction coordinate. A clear parabolic trend can be observed, nearly symmetrical for both forming bonds. The Cartesian coordinates of the key structures along pathway B are provided in the Supplementary Materials.

The progress indices (Δl) for the forming bonds differ for most TSs, indicating asynchronous bond formation In TS1, l_C1–C6_ = 0.70, while l_C4−C7_ = 0.12. This disparity indicates that the C1–C6 bond forms significantly earlier than the C4–C7 bond, suggesting pronounced asynchronicity. Similarly, in TS2, l_C1−C6_ = 0.97, while l_C4−C7_ = 0.00, showing that the C1–C6 bond is almost completely formed, whereas the C4–C7 bond has not yet begun to develop. Higher Δl values indicate greater asynchronicity.

### 2.2. Evidence of the Zwitterionic Nature of the Acyclic Intermediate

To gain a deeper understanding of the structure and properties of the zwitterionic form, we decided to investigate this species in detail. In particular, we aimed to elucidate how intramolecular charge separation influences its geometry, stability, and electronic distribution. To this end, we performed a series of quantum chemical analyses, including the Electron Localization Function (ELF), Natural Population Analysis (NPA), and Molecular Electrostatic Potential (MEP) mapping. These complementary approaches allowed us to characterize the electron density distribution, identify regions of charge accumulation and depletion, and assess the electrostatic features governing the stability of the zwitterionic form.

#### 2.2.1. Analysis of the Electronic Structure of the Zwitterion Based on NPA and MEP

NPA and MEP analyses are essential tools for understanding the electronic structure of molecular systems. NPA provides quantitative information about atomic charges and charge delocalization, while MEP visualizes the spatial distribution of electrostatic potential. Together, these methods enable precise identification of nucleophilic and electrophilic regions, offering valuable insight into charge separation, intramolecular interactions, and the overall stabilization of zwitterionic intermediates.

**Figure 11:**
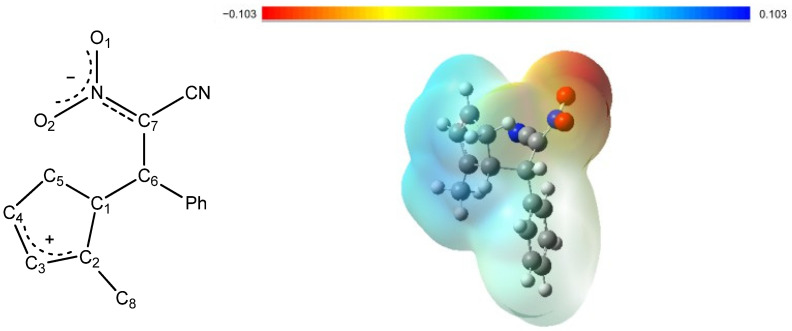
General structure of the zwitterion ZW and its DFT-calculated molecular electrostatic potential (MEP) map. In the MEP representation, red indicates electron-rich regions, while blue corresponds to electron-poor areas, highlighting the charge separation within the zwitterionic intermediate.

The NPA charge distribution (Figure 11, Table 7) clearly demonstrates the zwitterionic nature of the intermediate. The carbon and hydrogen atoms forming the cyclopentadienyl ring carry an overall positive charge, indicating electron deficiency within this fragment. In contrast, the negative charge is predominantly localized on the nitro group, as evidenced by the highly negative partial charges on the oxygen atoms (O1, −0.522 e; O2, −0.548 e). This separation of charge gives rise to a distinct dipole moment and reflects the polarization characteristic of the zwitterionic state. In the atomic charge analysis, the sum of all atomic charges in the five-membered ring, including C8 and its attached hydrogens, amounts to 0.825 e, confirming that the positive charge is predominantly localized within this fragment. The MEP map confirms this distribution, showing regions of positive electrostatic potential over the ring and pronounced negative potential around the nitro substituent, corresponding to the electrophilic and nucleophilic regions of the molecule, respectively.

#### 2.2.2. The Electron Localization Function (ELF)

The Electron Localization Function (ELF) analysis [33] is a useful tool for visualizing the distribution and localization of electron pairs in molecules. In the case of zwitterionic systems, ELF provides insight into how electron density is partitioned between regions of positive and negative charge, revealing the degree of covalency and polarization within the structure. This allows for a clear distinction between bonding, non-bonding, and delocalized regions, offering a deeper understanding of the electronic factors responsible for the stability of the zwitterionic intermediate.

**Figure 12:**
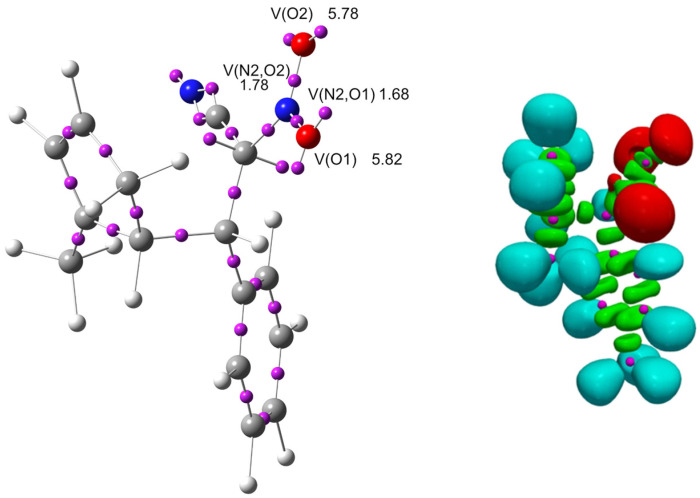
ωB97X-D/6-311g(d): ELF localization domains of ZW represented at an isosurface value of ELF = 0.75. ELF basin attractor positions, together with the most representative valence basin populations.

ELF localization analysis (Figure 12, Table 8) revealed that the largest populations of bonding basins are observed for the C3–C4 (2.88 e) and C7–N2 (2.79 e) bonds, indicating an increased electron density between these atoms and suggesting partial multiple-bond character or significant electron delocalization in these regions. The lone pairs on oxygen atoms are strongly localized (O1: 2.86–2.96 e; O2: 2.85–2.93 e), confirming that oxygen atoms are the main centers of negative charge in the studied zwitterion. Nitrogen atom N1 exhibits a high lone-pair basin population (3.34 e), which indicates substantial localization of electron density at this site and is relevant for describing intramolecular electrostatic interactions. A key piece of evidence supporting the zwitterionic character of ZW is the absence of ELF basins at C4, whereas C7 exhibits two basins containing 0.61 and 0.53 electrons (1.14 e in total). This distribution is consistent with a zwitterionic intermediate; if the species were radical, ELF basins would be present at both C4 and C7.

#### 2.2.3. Analysis of the Non-Covalent Interactions in the Zwitterion Based on NCI

The NCI analysis is performed to visualize and characterize weak non-covalent interactions that are not easily captured through conventional geometric or energetic criteria. By analyzing the sign(λ_2_)ρ function and corresponding isosurfaces, one can distinguish between attractive, neutral, and repulsive regions of electron density. This method provides valuable insight into the balance of van der Waals, electrostatic, and steric effects that govern the stability and reactivity of molecular systems. In the case of zwitterionic species, NCI analysis helps to identify the intramolecular interactions responsible for charge delocalization and overall stabilization of the intermediate.

**Figure 13:**
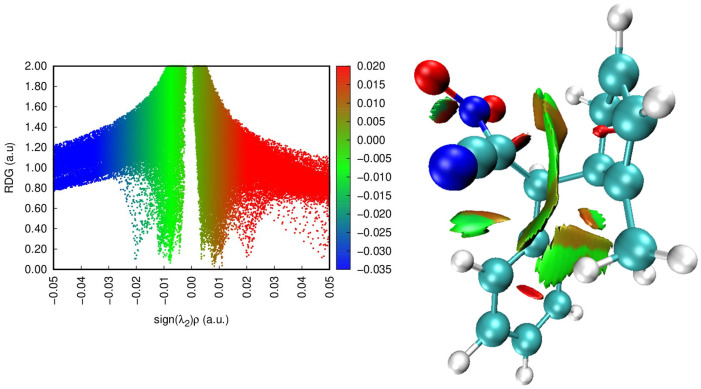
RDG scatter plots (**left**) and non-covalent interactions (NCI) plots (**right**) of ZW**_B_**. Colour online: blue represents strong attractive interactions, green indicates van der Waals interactions and red indicates repulsive/steric interactions.

The NCI analysis (Figure 13) of the zwitterionic intermediate reveals the presence of several regions of non-covalent interactions (Figure 13). The turquoise isosurfaces in the region of sign(λ_2_)ρ ≈ −0.02 a.u. correspond to weak attractive van der Waals interactions, while the green ones (around −0.01 a.u.) indicate slightly weaker dispersion contacts. In contrast, the red isosurfaces (0.01–0.02 a.u.) represent steric repulsion zones. These features are mainly located near the nitro group, reflecting the polarization and charge separation characteristic of the zwitterionic structure. The observed attractive regions suggest intramolecular stabilization of the zwitterionic intermediate through weak contacts involving the NO_2_ group.

### 2.3. Reaction Mechanism Elucidated by Bonding Evolution Theory (BET)

To gain detailed insight into the reaction mechanism and the evolution of chemical bonds within the zwitterionic system, we performed a Bonding Evolution Theory (BET) analysis [34]. This approach allows for a precise characterization of bond formation and cleavage along the reaction pathway, providing a clear picture of electron density redistribution during the transformation. By applying BET, we aimed to identify key stages in the reaction, understand the sequence of bond-making and bond-breaking events, and correlate these changes with the overall stability and reactivity of the zwitterionic species.

**Figure 14:**
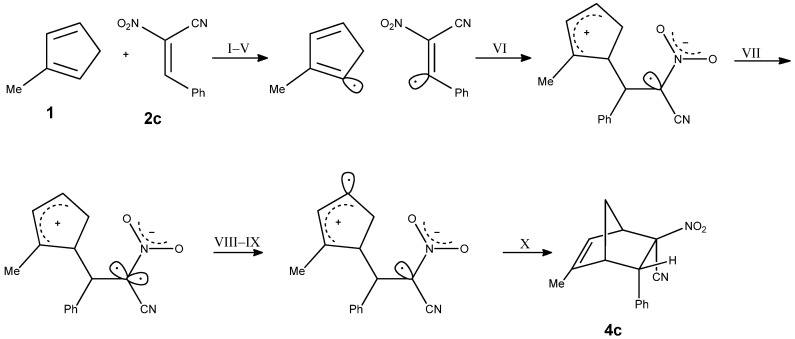
Simplified representation of the molecular mechanism of the reaction between **1** and **2c** using ELF−based Lewis structures.

The analysis of the ELF valence basin population changes along the IRC of the 42CA reaction between 1 and 2c shows that this reaction can be topologically characterized by 10 distinct phases of bond evolution for the reaction, clearly demonstrating a highly asynchronous and polar cycloaddition mechanism (Figure 14, Table 9).

At the early stages of the process (points 1–3), the disynaptic basins V(C1, C2) and V(C3, C4) increase in population, which corresponds to the initial strengthening of the σ-framework during reactant approach. Subsequently, both basins undergo a marked decrease, reflecting the rupture of the original C–C bonds. Subsequently, monosynaptic basins appear at C1 and C6, which then merge into a disynaptic basin V(C1–C6), corresponding to the formation of the C–C bond in the zwitterionic structure (ZW**_B_**). In the following steps, monosynaptic basins at C4 and C7 are formed first, which subsequently evolve into the disynaptic basin V(C4–C7), leading to the formation of the nitronorbornene structure. The process is polar and highly asynchronous, proceeding through a zwitterionic intermediate.

The basins associated with the aromatic ring (V(C10–C15)) remain nearly constant (2.7–2.8 e), confirming that the phenyl substituent does not undergo significant electronic reorganization and mainly serves as an electron-withdrawing stabilizer.

Throughout the reaction coordinate, a systematic redistribution of electron density occurs within the π-system and the nitro group. The evolution of V(C7, N2) and V(C9, N1) basins clearly indicates charge polarization toward the nitron fragment, supporting the zwitterionic character of the intermediate.

*Phase I* The key event in this stage is the merging of two disynaptic basins V(C6, C7) and V′(C6, C7), initially integrating 1.80 e and 1.70 e, respectively. As a result, a single, larger basin is formed with a population of 3.49 e.

*Phase II* At this stage, the disynaptic basins V(C1, C2) and V′(C1, C2) merge, increasing their populations from 1.75 e and 1.53 e to 3.24 e. The auxiliary basins V′(C1, C2) vanish, indicating the formation of stronger and more localized bonding interactions.

*Phase III* At this stage, the disynaptic basins V(C2, C3) and V′(C2, C3) merge, increasing their populations from 1.69 e and 1.56 e to 3.11 e. The auxiliary basins V′(C3, C4) vanish. The increase in V(C7, N2) (from 2.36 e to 2.65 e) suggests a change in the bonding regime in this region of the structure.

*Phase IV* A two newly formed monosynaptic basins with a population of 0.34 e and 0.09 e appear, indicating that atoms C1 and C6 begin to exhibit a certain degree of non-bonding electron density. This correlates with a slight decrease in V(C1, C2) (from 3.11 e to 2.77 e) and V(C1, C5) (from 2.01 e to 1.99 e), reflecting a partial weakening of bonding around C1.

*Phase V* The monosynaptic basins V(C1) and V(C6), formed in the previous phase, disappear, and a new disynaptic basin V(C1, C6) is formed from their merging electron densities. Additionally, a new monosynaptic basin with a total electron population of 0.39 e appears. A noticeable decrease in V(C6, C7) (from 3.44 e to 3.01 e) suggests that part of the C6–C7 bonding electron density has been redistributed into the new basins V(C6), V(C7), and V(C1, C6).

*Phase VI* A second monosynaptic basin V’(C7) with a population of 0.47 e appears, accompanied by an increase in V(C1, C6) (from 0.62 e to 0.99 e) and a further decrease in V(C6, C7) (from 3.01 e to 2.39 e). Atom C7 seems to exhibit two independent regions of non-bonding electron density, and the C6–C7 bond continues to weaken, favoring the formation of a new C1–C6 interaction.

*Phase VII* The V′(C7) basin disappears completely, indicating the loss of one of the previously separated regions of electron localization around atom C7. Simultaneously, the population of the V(C7) basin increases significantly, suggesting that the electron density formerly associated with V′(C7) has been transferred to V(C7).

*Phase VIII* A new monosynaptic basin V(C4) (0.10 e) appears, indicating a partial localization of electron density at the C4 atom. This newly formed basin reflects a transient accumulation of electronic charge that precedes the formation of the C4–C7 bond. The emergence of V(C4) thus marks an intermediate stage of the electronic reorganization, where the weakening of the former C3–C4 interaction facilitates the redistribution of electron density toward the forthcoming bonding region between C4 and C7.

*Phase IX* The disynaptic basin splits into two distinct basins, V(C2, C3) and V′(C2, C3), whose populations decrease from 3.10 e to 1.56 e and 1.58 e, respectively. This division indicates a redistribution of the electron density within the C2–C3 bonding region, suggesting a partial weakening or polarization of this bond.

*Phase X* A new disynaptic basin V(C4, C7) with a population of 1.11 e appears, marking the formation of a new C–C bond between atoms C4 and C7. This process is accompanied by a redistribution of electron density from the previously existing monosynaptic basins V(C4) and V(C7), which disappear at this stage. The vanishing of these monosynaptic basins indicates that the non-bonding electron density localized on C4 and C7 has been fully incorporated into the new bonding region, confirming the establishment of a stable σ-type C–C.

### 2.4. Evaluation of Substituent Effects on the Mechanistic Pathways

To verify whether the reaction mechanism established for the parent system can be considered general, we extended our study to include a substituent effect analysis. For this purpose, a series of conjugated nitroalkenes bearing para-substituted phenyl rings were investigated. The substituents were selected to cover a broad range of electronic effects as defined by their Hammett σ parameters, encompassing both electron-donating (-OMe, -Me) and electron-withdrawing (-F, -Cl, -NO_2_) groups.

The analysis of the corresponding potential energy surfaces revealed that the overall mechanistic pattern remains preserved across the entire substituent series. In all cases, the reaction proceeds via formation of a pre-reactive molecular complex, followed by a single or double transition-state pathway, depending on the specific reaction channel (A–D). However, quantitative differences were observed in the relative energies of the critical points, indicating that the electronic nature of the substituent subtly modulates the activation barriers and the stability of intermediates.

The relative thermodynamic parameters calculated for the stationary points along path A of the cycloaddition reaction of **1** with **2a**–**f** (Table 10). The substituent effect on the reaction profile is clearly visible in the first step, where the Gibbs free energy barriers (ΔG^≠^ for MC → TS1) decrease from 15.4 kcal·mol^−1^ for the electron-donating OMe group to 11.2 kcal·mol^−1^ for the strongly electron-withdrawing NO_2_ group. This trend indicates that electron-accepting substituents facilitate the initial cycloaddition step by stabilizing the transition state. In contrast, the second-step barriers (INT → TS2) remain nearly constant (~15 kcal·mol^−1^) across all derivatives, suggesting a less pronounced substituent influence. The relative stability of the products (PR) follows the order NO_2_ > Cl > H ≈ F > Me > OMe, in agreement with the electron-withdrawing strength of the substituents. Entropic contributions are similar for all systems (ΔS ≈ −50 to −55 cal·mol^−1^·K^−1^), reflecting comparable structural reorganization upon cycloaddition.

In contrast to path A, this route (path B) proceeds through a zwitterionic intermediate (ZW) stabilized to various extents depending on the substituent at the phenyl ring. The first activation barrier (MC → TS) gradually decreases with increasing electron-withdrawing strength of the substituent, from 17.3 kcal·mol^−1^ for OMe to only 13.2 kcal·mol^−1^ for NO_2_, indicating that electrophilic activation of the dienophile favors the polar transition state. The zwitterionic intermediates are notably stabilized in the presence of electron-accepting substituents (ΔG(ZW) = 15.6 for Ome and 10.6 kcal·mol^−1^ for NO_2_), supporting a polar, two-step mechanism. The relative Gibbs free energies of the products follow the same trend as in path A, decreasing in the order OMe < Me < F < H < Cl < NO_2_. The NO_2_-substituted system exhibits the lowest energy barriers and the most stable product, confirming that path B is strongly favored for electron-deficient derivatives. Entropic effects remain similar across the series (ΔS ≈ −50 cal·mol^−1^·K^−1^), reflecting comparable degrees of structural ordering during the reaction progress.

Similarly to path A, this mechanism proceeds via a two-step process involving an intermediate (INT) (path C). The Gibbs free energy barriers of the first step (MC → TS1) range from 16.9 kcal·mol^−1^ for the OMe derivative to 13.2 kcal·mol^−1^ for the NO_2_-substituted system, showing a clear substituent effect consistent with the electron-withdrawing strength at the phenyl ring. Electron-accepting groups significantly lower both activation barriers and stabilize the intermediate and final product. The second transition state (INT → TS2) shows slightly lower and more uniform barriers (ΔG^≠^ ≈ 17–14 kcal·mol^−1^), suggesting a relatively similar energy demand for the ring closure step across all derivatives. The product stability follows the same trend as in paths A and B, increasing with the electron-withdrawing character of the substituent (OMe < Me < F < H < Cl < NO_2_). Overall, the NO_2_-substituted system exhibits the lowest free energies throughout the entire reaction profile, indicating a highly favorable reaction pathway. Entropic variations (ΔS ≈ −50 cal·mol^−1^·K^−1^) remain small, confirming that the reaction proceeds with comparable degrees of molecular organization for all substituents.

Unlike paths A–C, this mechanism proceeds through a transition state without the formation of an intermediate (path D). The Gibbs free energy barriers (MC → TS) systematically decrease with increasing electron-withdrawing ability of the substituent, from 18.6 kcal·mol^−1^ for the OMe derivative to 13.8 kcal·mol^−1^ for the NO_2_-substituted system. This trend clearly indicates that electron-accepting groups promote a more facile, concerted cycloaddition process. The relative stability of the products again follows the order OMe < Me < F < H < Cl < NO_2_, in line with their electronic nature. Among all derivatives, the NO_2_-substituted system exhibits both the lowest activation barrier and the most stable product, suggesting that the D path is thermodynamically and kinetically favored under polar conditions for electron-deficient substrates. Entropic changes remain consistent across the series (ΔS ≈ −50 cal·mol^−1^·K^−1^), implying similar degrees of molecular organization in the transition states.

Comparison of the four examined reaction pathways (A–D) indicates that the substituent at the phenyl ring has a pronounced influence on both the mechanism and energetics of the cycloaddition. For all cases, the product stability increases with the electron-withdrawing strength of the substituent (OMe < Me < F < H < Cl < NO_2_). Overall, the NO_2_-substituted system exhibits the lowest Gibbs free energies and the smallest barriers across all pathways, confirming that electron-deficient derivatives react more readily via a polar, nearly concerted mechanism. Electron-withdrawing groups were found to lower the activation energy, thereby facilitating the cycloaddition process, while electron-donating substituents produced the opposite effect. These observations are consistent with the general trend predicted by the Hammett correlation, confirming that the reaction is governed primarily by electronic rather than steric factors. Consequently, the investigated mechanism can be regarded as general and robust with respect to substituent-induced perturbations in the electronic structure of the nitroalkene component.

## 3. Computational Details

All calculations were performed using the GAUSSIAN 16 software package [35], employing the ωB97X-D functional [36] in combination with the 6-311G(d) basis set [37,38,39]. Molecular geometries were visualized using GaussView 6.0 [40]. Thermodynamic and kinetic parameters were calculated at the ωB97X-D/6-311G(d) level of theory in the presence of various solvents, simulated using the Polarizable Continuum Model (PCM) [41,42]. Calculations were carried out for the gas phase and for selected solvents of different polarities (e.g., acetone, acetonitrile, and toluene) to evaluate solvent effects on reaction energetics and mechanism. For the optimized structures, thermochemical data were derived for a temperature of T = 298 K and a pressure of *p* = 1 atm, based on vibrational analysis.

All reactants, intermediates, and products were optimized using the Berny algorithm [43,44], ensuring convergence to stationary points on the potential energy surface. Transition state (TS) geometries were obtained through the QST2 (Quadratic Synchronous Transit) procedure, followed by harmonic frequency analyses to verify their nature. Minima were confirmed by the presence of exclusively positive Hessian eigenvalues, whereas each transition state was characterized by a single imaginary frequency corresponding to the reaction coordinate, as validated through inspection of the respective vibrational mode. The connectivity between each transition state and its neighboring minima was subsequently verified by performing Intrinsic Reaction Coordinate (IRC) calculations in both forward and reverse directions.

The Global Electron Density Transfer (GEDT) [45] values were obtained from Natural Population Analysis (NPA) [46,47]. GEDT(f) was evaluated as the atomic charge qf associated with fragment (f) at the transition states. The distance-based progress indices (l) were computed following the standard procedure outlined in [48].

$$l_{X-Y}=1-\frac{r_{X-Y}^{TS}-r_{X-Y}^{P}}{r_{X-Y}^{P}}$$

where r^TS^_X−Y_ is the distance between the reaction centers X and Y in the TS and r^P^_X−Y_ is the same distance in the corresponding product.

Topological analyses of the Electron Localization Function (ELF) [33,49,50] were carried out with the TopMod 09 program [51], based on monodeterminantal wavefunctions computed on a grid with a spatial resolution of 0.1 atomic units. The molecular structures, together with three-dimensional representations of radical cations, radical anions, and ELF basin attractors, were visualized using GaussView 6.0. For spatial inspection, ELF localization domains were further generated in ParaView 5.13.3 at an isovalue of 0.75 atomic units. The NCI studies were performed with Multiwfn 3.7 [52] software.

## 4. Conclusions

We investigated the cycloaddition reaction of cyclopentadiene with conjugated nitroalkenes. In a nonpolar medium such as toluene, the reaction pathways A and C proceed through a pre-reactive molecular complex (MC), two transition states, and a heterocyclic nitro intermediate. In contrast, pathways B and D involve the formation of the pre-reactive complex (MC) followed by a single transition state leading directly to the norbornene reaction product. A moderate increase in solvent polarity (as in acetone) does not lead to qualitative changes in the reaction’s energy profile. The overall reaction mechanism remains the same—all four pathways proceed via an identical mechanistic pattern. Quantitatively, the profile of the critical points along the reaction coordinate differs somewhat, but not to an extent that would alter the reaction mechanism. Significant mechanistic changes occur upon introducing a more polar solvent, such as methanol. While the description of pathways A, C, and D remains quantitatively unaffected, the mechanism along pathway B undergoes a fundamental transformation. Pathway B proceeds through a solvent-dependent mechanism. In nonpolar solvents, it follows a concerted, one-step route via a single transition state leading directly to the product. In contrast, in polar solvents, the reaction adopts a stepwise course involving a zwitterionic intermediate.

Subsequently, we examined the influence of solvent polarity in greater detail. A further increase in solvent polarity (acetonitrile, water) promotes a decrease in the energies of the critical points along the reaction coordinate, without altering the mechanistic character of the individual reaction pathways.

Additionally, NPA, MEP, and NCI analyses were performed for the zwitterionic intermediate to unambiguously confirm its polar nature and the presence of distinct charge-separated regions, thereby validating its zwitterionic character.

The BET analysis confirms that the studied cycloaddition path B proceeds through a polar, highly asynchronous, and stepwise mechanism involving a zwitterionic intermediate. The sequential formation and merging of disynaptic and monosynaptic basins clearly illustrate the gradual transformation of the σ-framework and the redistribution of electron density during bond formation and cleavage. The reaction pathway is characterized by an early polarization toward the nitro fragment, followed by the stepwise formation of new C–C bonds (C1–C6 and C4–C7). The aromatic substituent remains electronically inert, serving mainly as a stabilizing electron-withdrawing group. Overall, the process demonstrates a well-defined electronic reorganization consistent with a zwitterionic mechanism of nitronorbornene formation.

Finally, in order to determine whether the investigated mechanism is of a general nature, I performed a substituent effect analysis by introducing a series of para-substituents with known Hammett σ constants into the phenyl ring of the conjugated nitroalkene. Electron-withdrawing substituents decrease the activation barriers, thus promoting the cycloaddition, whereas electron-donating groups lead to an increase in the energy demand of the process. This behavior aligns well with the tendencies predicted by Hammett correlations, indicating that electronic effects dominate over steric influences in controlling the reaction course. Therefore, the proposed mechanism appears to be general and resilient to electronic perturbations introduced by various substituents on the nitroalkene moiety.

## Figures and Tables

**Table 1:** Electronic properties of the reactants: methylcyclopentadiene (**1**) and E-2-aryl-1-cyano-1-nitroethenes (**2a**–**f**) [18].

	Substituent		Global Properties	
	R	σ_p_	ω	N
**1**			0.52	3.46
**2a**	OMe	−0.27	1.74	2.68
**2b**	Me	−0.17	1.86	2.22
**2c**	H	0.00	1.95	1.95
**2d**	F	0.06	1.98	1.98
**2e**	Cl	0.23	2.06	1.99
**2f**	NO_2_	0.78	2.49	1.27

**Table 2:** ωB97X-D/6-311g(d). Relative enthalpies (ΔH in kcal·mol^−1^), entropies (ΔS in cal·mol^−1^ K^−1^) and Gibbs free energies (ΔG in kcal·mol^−1^) of the stationary points involved in the CA reaction of 1 with 2C, path A. Energies are reported relative to the separated reactants (1 + 2c).

	Toluene			Acetone			Methanol			Acetonitrile			Nitromethane			Water		
	ΔH	ΔG	ΔS	ΔH	ΔG	ΔS	ΔH	ΔG	ΔS	ΔH	ΔG	ΔS	ΔH	ΔG	ΔS	ΔH	ΔG	ΔS
**MC_A_**	−9.3	2.8	−40.4	−8.5	3.1	−38.9	−8.4	3.1	−38.6	−8.4	3.0	−38.5	−8.4	3.0	−38.5	−8.4	3.1	−38.4
**TS1_A_**	3.3	17.9	−49.0	1.9	16.8	−50.0	1.8	16.5	−49.5	1.8	16.5	−49.4	1.8	16.5	−49.4	1.7	16.2	−48.9
**INT_A_**	−20.5	−3.1	−58.3	−20.2	−3.6	−55.7	−20.3	−3.8	−55.3	−20.3	−3.8	−55.3	−20.3	−3.8	−55.2	−20.3	−3.9	−54.9
**TS2_A_**	−0.9	16.2	−57.4	−4.7	11.8	−55.1	−4.9	11.5	−54.9	−4.9	11.4	−54.8	−4.9	11.4	−54.8	−5.1	11.2	−54.6
**PR_A_**	−26.7	−10.3	−54.9	−26.1	−10.1	−53.6	−26.1	−10.1	−53.5	−26.1	−10.1	−53.4	−26.1	−10.1	−53.4	−26.1	−10.2	−53.4

**Table 3:** ωB97X-D/6-311g(d). Relative enthalpies (ΔH in kcal·mol^−1^), entropies (ΔS in cal·mol^−1^ K^−1^) and Gibbs free energies (ΔG in kcal·mol^−1^) of the stationary points involved in the CA reaction of 1 with 2C, path C. Energies are reported relative to the separated reactants (1 + 2c).

	Toluene			Acetone			Methanol			Acetonitrile			Nitromethane			Water		
	ΔH	ΔG	ΔS	ΔH	ΔG	ΔS	ΔH	ΔG	ΔS	ΔH	ΔG	ΔS	ΔH	ΔG	ΔS	ΔH	ΔG	ΔS
**MC_C_**	−9.8	2.6	−41.5	−8.9	3.4	−41.1	−8.9	3.3	−40.7	−8.9	3.3	−40.7	−8.9	3.3	−40.7	−8.8	3.2	−40.4
**TS1_C_**	4.6	20.4	−52.8	3.5	18.8	−51.3	3.5	18.7	−51.1	3.4	18.7	−51.0	3.4	18.6	−51.0	3.4	18.5	−50.8
**INT_C_**	−20.4	−3.9	−55.4	−11.7	4.6	−54.4	−20.3	−4.3	−53.7	−20.3	−4.4	−53.6	−20.3	−4.4	−53.6	−20.4	−4.5	−53.2
**TS2_C_**	3.0	19.7	−56.1	7.5	23.9	−54.9	0.4	16.4	−53.7	0.4	16.4	−53.7	0.4	16.4	−53.7	0.2	16.2	−53.7
**PR_C_**	−26.4	−9.8	−55.6	−18.5	−2.4	−54.1	−25.5	−9.2	−54.9	−25.5	−9.2	−54.8	−25.5	−9.2	−54.8	−25.5	−9.3	−54.5

**Table 4:** ωB97X-D/6-311g(d). Relative enthalpies (ΔH in kcal·mol^−1^), entropies (ΔS in cal·mol^−1^ K^−1^) and Gibbs free energies (ΔG in kcal·mol^−1^) of the stationary points involved in the CA reaction of 1 with 2C, path D. Energies are reported relative to the separated reactants (1 + 2c).

	Toluene			Acetone			Methanol			Acetonitrile			Nitromethane			Water		
	ΔH	ΔG	ΔS	ΔH	ΔG	ΔS	ΔH	ΔG	ΔS	ΔH	ΔG	ΔS	ΔH	ΔG	ΔS	ΔH	ΔG	ΔS
**MC_D_**	−8.6	3.3	−40.1	−0.6	10.6	−37.8	−7.9	2.9	−36.1	−7.9	3.3	−37.3	−7.8	3.5	−38.0	−7.8	3.2	−36.9
**TS_D_**	6.7	22.3	−52.3	5.4	20.0	−49.2	5.2	19.6	−48.2	5.2	19.4	−47.7	5.2	19.4	−47.5	5.1	19.6	−48.5
**PR_D_**	−25.9	−9.4	−55.4	−18.0	−1.5	−55.3	−25.1	−8.7	−55.0	−25.0	−8.7	−54.9	−25.0	−8.7	−54.9	−25.0	−8.7	−54.6

**Table 5:** ωB97X-D/6-311g(d). Relative enthalpies (ΔH in kcal·mol^−1^), entropies (ΔS in cal·mol^−1^ K^−1^) and Gibbs free energies (ΔG in kcal·mol^−1^) of the stationary points involved in the CA reaction of 1 with 2C, path B. Energies are reported relative to the separated reactants (1 + 2c).

	Toluene			Acetone			Metanol			Acetonitrile			Nitrometan			Water		
	ΔH	ΔG	ΔS	ΔH	ΔG	ΔS	ΔH	ΔG	ΔS	ΔH	ΔG	ΔS	ΔH	ΔG	ΔS	ΔH	ΔG	ΔS
**MC_B_**	−9.5	2.6	−40.5	−1.5	9.7	−37.5	−8.8	3.1	−39.7	−8.8	3.0	−39.4	−8.8	3.0	−39.5	−8.7	3.0	−39.4
**TS_B_**	4.5	20.0	−52.0	3.0	18.4	−51.8	2.9	18.2	−51.4	2.9	18.2	−51.4	2.9	18.2	−51.4	2.8	18.0	−51.1
**ZW_B_**							−1.1	13.8	−49.9	−1.1	13.7	−49.8	−1.1	13.7	−49.8	−1.4	13.3	−49.4
**TS2_B_**							−0.8	14.6	−51.6	−1.0	15.2	−54.3	−1.0	15.2	−54.3	−1.5	13.8	−51.3
**PR_B_**	−26.0	−8.9	−57.4	−18.0	−1.3	−56.1	−25.4	−9.1	−54.6	−25.3	−9.1	−54.5	−25.3	−9.1	−54.5	−25.3	−9.1	−54.4

**Table 6:** The key parameters of the critical structure parameters of reaction of 1 and 2c according to ωB97X-D/6-311g(d) calculations.

	r_C1–C6_ [Å]	l	r_C4–C7_ [Å]	l	Δl	GEDT [e]
**MC_B_**	3.25		3.38			
**TS1_B_**	2.05	0.70	2.98	0.12	0.58	0.43
**ZW_B_**	1.615		3.24			
**TS2_B_**	1.625	0.97	3.17	0.00	0.97	0.79
**PR_B_**	1.57		1.58			

**Table 7:** NPA (Natural Population Analysis) atomic charges calculated at the ωB97X-D/6−311g(d) level for selected atoms in the studied systems.

Atom	Charge	Atom	Charge
C1	−0.262	C8	−0.651
H1	0.265	H8	0.274
C2	0.326	H8′	0.243
C3	−0.299	H8″	0.242
H3	0.252	C6	−0.217
C4	0.120	C7	−0.171
H4	0.231	H7	0.272
C5	−0.456	N1	0.503
H5	0.262	O1	−0.522
H5′	0.278	O2	−0.548

**Table 8:** The most significant ELF valence basin populations N for ZW**_B_**, given an average number of electrons [e].

ELF Basin	N[e]	ELF Basin	N[e]
V(C2, C8)	2.1	V′(C7)	0.53
V(C1, C2)	2.2	V(C7, N2)	2.79
V(C2, C3)	2.63	V(C9, N1)	1.94
V(C1, C6)	1.58	V′(C9, N1)	2.35
V(C1, C5)	1.89	V(N1)	3.34
V(C3, C4)	2.88	V(N2, O1)	1.68
V(C4, C5)	2.08	V′(N2, O2)	1.78
V(C6, C10)	2.06	V(O1)	2.96
V(C6, C7)	2.08	V′(O1)	2.86
V(C7, C9)	2.49	V(O2)	2.93
V(C7)	0.61	V′(O2)	2.85

**Table 9:** Method ELF valence basin populations of the most relevant structures obtained from the IRC path along the reaction path C of the CA reaction between **1** and **2c**.

Point	1	2	3	4	5	6	7	8	9	10	11
V(C1, C2)	1.72	1.75	3.24	3.11	2.77	2.66	2.48	2.24	2.16	2.15	2.03
V′(C1, C2)	1.63	1.53									
V(C2, C3)	2.27	2.3	2.32	2.43	2.46	2.49	2.56	2.79	3.1	1.56	1.71
V’(C2, C3)										1.58	1.79
V(C1, C8)	2.03	2.04	2.04	2.06	2.06	2.07	2.07	2.08	2.08	2.07	2.05
V(C10, C11)	2.71	2.71	2.72	2.76	2.77	2.77	2.79	2.78	2.78	2.78	2.77
V(C10, C12)	2.7	2.71	2.71	2.74	2.75	2.75	2.76	2.82	2.83	2.83	2.83
V(C11, C13)	2.78	2.78	2.78	2.76	2.76	2.76	2.75	2.78	2.78	2.78	2.78
V(C12, C14)	2.81	2.82	2.82	2.82	2.81	2.81	2.81	2.77	2.76	2.76	2.77
V(C13, C15)	2.73	2.74	2.74	2.76	2.76	2.76	2.77	2.75	2.75	2.75	2.75
V(C14, C15)	2.73	2.73	2.72	2.73	2.73	2.73	2.74	2.76	2.76	2.76	2.76
V(C1)					0.34						
V(C1, C5)	2.02	2.03	2.03	2.01	1.99	1.98	1.94	1.84	1.82	1.82	1.83
V(C1, C6)						0.62	0.99	1.53	1.65	1.66	1.82
V(C4)									0.1	0.11	
V(C3, C4)	1.69	1.69	1.69	3.11	3.08	3.06	2.98	2.76	2.47	2.45	1.97
V′(C3, C4)	1.61	1.57	1.56								
V(C4, C5)	2.01	2.02	2.02	2.04	2.05	2.06	2.06	2.09	2.04	2.04	1.83
V(C4, C7)											1.89
V(C6)					0.09						
V(C6, C10)	2.31	2.3	2.3	2.26	2.24	2.22	2.15	2.07	2.06	2.06	2.08
V(C6, C7)	1.8	3.49	3.49	3.53	3.44	3.01	2.39	2.46	2.2	2.18	1.95
V′(C6, C7)	1.7										
V(C7)						0.39	0.45	0.67	0.9	0.93	
V′(C7)							0.47				
V(C7, C9)	2.4	2.41	2.41	2.43	2.44	2.44	2.45	2.48	2.44	2.43	2.25
V(C7, N2)	2.32	2.35	2.36	2.65	2.7	2.72	2.77	2.81	2.57	2.55	2.44
V(C9, N1)	1.64	1.66	1.66	1.72	1.72	1.73	1.75	1.92	1.99	2.0	2.59
V′(C9, N1)	2.8	2.76	2.76	2.66	2.65	2.64	2.6	2.41	2.42	2.41	1.9
V(N1)	3.19	3.21	3.22	3.26	3.27	3.27	3.29	3.31	3.24	3.24	3.18
V(N2, O1)	1.84	1.83	1.82	1.76	1.75	1.74	1.72	1.7	1.78	1.78	1.87
V(N2, O2)	2.2	2.17	2.17	1.9	1.85	1.83	1.8	1.79	1.99	1.98	2.02
V(O1)	2.84	2.85	2.87	2.9	2.91	2.91	2.93	2.93	2.87	2.86	2.79
V′(O1)	2.81	2.82	2.82	2.84	2.84	2.85	2.85	2.86	2.84	2.84	2.82
V(O2)	2.8	2.81	2.81	2.84	2.85	2.86	2.88	2.88	2.84	2.83	2.77
V′(O2)	2.81	2.82	2.83	2.84	2.85	2.86	2.86	2.86	2.84	2.84	2.82

**Table 10:** ωB97X-D/6-311G(d). Relative enthalpies (ΔH in kcal·mol^−1^), entropies (ΔS in cal·mol^−1^·K^−1^), and Gibbs free energies (ΔG in kcal·mol^−1^) of the stationary points involved in the CA reaction of **1** with **2a**–**f** calculated in nitromethane. Energies are reported relative to the separated reactants (**1** + **2a**–**f**).

	R	Path	Structure	ΔH	ΔG	ΔS
**2a**	OMe	A	MC	−8.2	2.7	−36.8
			TS1	3.8	18.1	−47.9
			INT	−17.6	−1.8	−53.1
			TS2	−2.0	13.8	−53.2
			PR	−23.3	−7.9	−51.6
		B	MC	−8.5	2.6	−37.0
			TS	4.9	19.9	−50.3
			ZW	1.6	15.6	−46.8
			TS2	1.8	17.7	−53.5
			PR	−22.6	−6.4	−54.3
		C	MC	−8.5	3.5	−40.2
			TS1	5.4	20.1	−49.3
			INT	−17.6	−2.1	−51.9
			TS2	2.9	18.5	−52.1
			PR	−22.8	−7.0	−52.9
		D	MC	−7.7	2.9	−35.4
			TS	7.5	21.5	−46.9
			PR	−22.6	−6.8	−52.9
**2b**	Me	A	MC	−7.8	2.0	−32.9
			TS1	3.1	15.6	−42.2
			INT	−18.7	−3.9	−49.5
			TS2	−3.2	11.3	−48.8
			PR	−25.0	−9.3	−52.8
		B	MC	−8.6	3.1	−39.5
			TS	4.1	17.1	−43.6
			ZW	0.5	13.7	−44.2
			TS2	0.5	13.6	−44.1
			PR	−24.4	−8.1	−54.5
		C	MC	−8.3	1.6	−33.4
			TS1	4.8	18.1	−44.8
			INT	−19.3	−3.6	−52.7
			TS2	2.0	16.4	−48.4
			PR	−23.9	−9.4	−48.5
		D	MC	−7.2	2.5	−32.3
			TS	6.6	19.6	−43.6
			PR	−24.1	−7.9	−54.3
**2c**	H	A	MC	−8.4	3.1	−38.6
			TS1	1.8	16.5	−49.5
			INT	−20.3	−3.8	−55.3
			TS2	−4.9	11.5	−54.9
			PR	−26.1	−10.1	−53.5
		B	MC	−8.8	3.1	−39.7
			TS	2.9	18.2	−51.4
			ZW	−1.1	13.8	−49.9
			TS2	−0.8	14.6	−51.6
			PR	−25.4	−9.1	−54.6
		C	MC	−8.9	3.3	−40.7
			TS1	3.5	18.7	−51.1
			INT	−20.3	−4.3	−53.7
			TS2	0.4	16.4	−53.7
			PR	−25.5	−9.2	−54.9
		D	MC	−7.9	2.9	−36.1
			TS	5.2	19.6	−48.2
			PR	−25.1	−8.7	−55.0
**2d**	F	A	MC	−8.4	2.9	−38.1
			TS1	2.0	16.6	−49.0
			INT	−20.1	−3.8	−54.6
			TS2	−4.6	11.8	−54.9
			PR	−25.9	−9.9	−53.5
		B	MC	−8.7	3.3	−40.3
			TS	3.0	18.2	−50.9
			ZW	−0.9	13.9	−49.7
			TS2	−1.0	14.2	−50.7
			PR	−25.3	−8.9	−55.0
		C	MC	−8.8	3.4	−41.1
			TS1	3.7	18.9	−51.0
			INT	−20.1	−4.3	−53.2
			TS2	0.6	16.6	−53.8
			PR	−25.4	−9.1	−54.7
		D	MC	−7.8	3.5	−37.8
			TS	5.5	20.1	−49.3
			PR	−25.1	−8.7	−54.8
**2e**	Cl	A	MC	−8.5	3.5	−40.2
			TS1	1.3	16.1	−49.6
			INT	−21.5	−4.6	−56.8
			TS2	−5.6	10.8	−54.9
			PR	−26.8	−10.7	−53.9
		B	MC	−9.0	3.0	−39.9
			TS	2.3	17.7	−51.5
			ZW	−1.8	13.3	−50.6
			TS2	−1.7	14.9	−55.6
			PR	−26.4	−10.0	−54.9
		C	MC	−8.9	3.5	−41.5
			TS1	3.0	18.2	−50.9
			INT	−21.0	−5.4	−52.3
			TS2	−0.3	15.8	−54.2
			PR	−26.3	−10.0	−54.9
		D	MC	−7.9	3.3	−37.8
			TS	4.7	19.4	−49.4
			PR	−26.0	−9.7	−54.9
**2f**	NO_2_	A	MC	−8.7	3.1	−39.5
			TS1	−0.3	14.3	−49.2
			INT	−23.0	−6.7	−55.0
			TS2	−8.1	8.2	−54.6
			PR	−29.1	−13.2	−53.6
		B	MC	−9.2	2.7	−40.0
			TS	0.6	15.9	−51.3
			ZW	−4.2	10.6	−49.4
			TS2	−3.7	12.1	−53.2
			PR	−28.4	−11.9	−55.4
		C	MC	−9.1	3.2	−41.3
			TS1	1.5	16.4	−50.2
			INT	−23.0	−7.1	−53.5
			TS2	−2.5	13.4	−53.0
			PR	−28.6	−12.3	−54.9
		D	MC	−8.2	3.4	−38.8
			TS	2.8	17.2	−48.0
			PR	−28.3	−11.9	−54.8

## Data Availability

The data that support the findings of this study are available from the corresponding author upon reasonable request.

## Supplementary Materials

The following supporting information can be downloaded at: https://www.mdpi.com/article/10.3390/molecules30244710/s1, Cartesian coordinates of the key structures along path B.
