# Supplementary material for: Zwitterionic Pathway in the Diels–Alder Reaction: Solvent and Substituent Effects from ωB97XD/6-311G(d) Calculations
Source: Molecules. 2025 Dec 9;30(24):4710. doi: 10.3390/molecules30244710 (PMC12735418; doi:10.3390/molecules30244710)
Supplement: Supplementary file 1 [file molecules-30-04710-s001.zip › molecules-4012828-supplementary.pdf]

SUPPLEMENTARY MATERIAL

Zwitterionic pathway in the Diels–Alder reaction: solvent and substituent effects from ωB97XD/6-311G(d) calculations

Agnieszka Łapczuk [1*]

[1] Cracow University of Technology, Faculty of Chemical Engineering and Technology, Department of Organic Chemistry and Technology, Warszawska 24, 31-155 Cracow, Poland
[*] Correspondence: agnieszka.lapczuk@pk.edu.pl

Cartesian coordinates of the key structures along path B

MC$_B$

Center Number	Atomic Number	Atomic Type	Coordinates (Angstroms) X	Y	Z
1	6	0	-0.102758	2.272824	0.043828
2	6	0	-0.383990	2.030713	-1.250276
3	6	0	-2.370659	1.882091	-0.015147
4	6	0	-1.353065	2.179015	0.810270
5	1	0	-1.418018	2.313771	1.883813
6	6	0	-1.853541	1.793009	-1.419440
7	1	0	-2.082383	0.836544	-1.901685
8	1	0	-2.312663	2.559222	-2.057017
9	6	0	0.062332	-0.960043	-0.617769
10	1	0	-0.174518	-1.071648	-1.671777
11	6	0	-1.000292	-1.127992	0.193095
12	1	0	0.329177	2.021089	-2.065925
13	1	0	-3.408755	1.743633	0.259247
14	6	0	1.228847	2.565926	0.655575
15	1	0	1.417910	1.906436	1.512569
16	1	0	2.037651	2.420855	-0.065570
17	1	0	1.276542	3.598056	1.027941
18	6	0	-1.048962	-0.978629	1.604747
19	7	0	-1.059570	-0.836533	2.747569
20	6	0	1.465154	-0.741797	-0.317339
21	6	0	2.017080	-0.787709	0.970685
22	6	0	2.318633	-0.543290	-1.413588
23	6	0	3.384170	-0.650506	1.147714
24	1	0	1.391693	-0.949304	1.838492
25	6	0	3.683917	-0.400291	-1.231834
26	1	0	1.901965	-0.508885	-2.414617
27	6	0	4.219590	-0.460397	0.050922
28	1	0	3.801477	-0.697475	2.146844
29	1	0	4.330766	-0.250906	-2.088414

30	1	0	5.289092	-0.359691	0.196877
31	7	0	-2.282171	-1.514276	-0.402760
32	8	0	-2.364157	-1.593485	-1.613484
33	8	0	-3.191956	-1.757257	0.365464

TS1$_B$

Center Number	Atomic Number	Atomic Type	Coordinates (Angstroms) X	Y	Z
1	6	0	-0.133157	2.068527	0.136397
2	6	0	-0.372675	1.476796	-1.107826
3	6	0	-2.382307	1.762661	0.028034
4	6	0	-1.369536	2.162781	0.842314
5	1	0	-1.464343	2.471702	1.875162
6	6	0	-1.862006	1.504242	-1.339721
7	1	0	-2.290964	0.638147	-1.842549
8	1	0	-2.091724	2.372828	-1.972683
9	6	0	-0.075133	-0.497205	-0.653366
10	1	0	-0.285866	-0.765935	-1.683656
11	6	0	-1.123131	-0.935490	0.183552
12	1	0	0.338420	1.554511	-1.921557
13	1	0	-3.427478	1.694029	0.301008
14	6	0	1.185598	2.480905	0.679721
15	1	0	1.370314	2.009194	1.649117
16	1	0	2.003819	2.232200	0.004559
17	1	0	1.187954	3.562513	0.847408
18	6	0	-1.141543	-0.893768	1.594153
19	7	0	-1.115026	-0.835998	2.748133
20	6	0	1.377560	-0.565857	-0.344392
21	6	0	1.908699	-0.704826	0.939925
22	6	0	2.261150	-0.484155	-1.427070
23	6	0	3.284224	-0.753014	1.131544
24	1	0	1.262053	-0.773070	1.804301
25	6	0	3.633720	-0.527147	-1.234960
26	1	0	1.866217	-0.384090	-2.433573
27	6	0	4.151092	-0.659103	0.049834
28	1	0	3.677913	-0.861695	2.136115
29	1	0	4.299097	-0.462002	-2.088670
30	1	0	5.223788	-0.692971	0.205436
31	7	0	-2.273875	-1.533227	-0.399157
32	8	0	-2.341472	-1.641630	-1.624004
33	8	0	-3.158804	-1.936229	0.349994

ZW$_B$

Center Number	Atomic Number	Atomic Type	Coordinates (Angstroms) X	Y	Z
1	6	0	-0.100652	2.078187	0.201924

2	6	0	-0.312702	1.320083	-1.033818
3	6	0	-2.330786	1.842015	0.011641
4	6	0	-1.325141	2.257073	0.851576
5	1	0	-1.451413	2.650552	1.850532
6	6	0	-1.803602	1.509583	-1.325721
7	1	0	-2.303924	0.677230	-1.818664
8	1	0	-1.934667	2.387621	-1.971664
9	6	0	-0.054030	-0.274483	-0.720917
10	1	0	-0.231526	-0.711531	-1.703915
11	6	0	-1.103845	-0.855297	0.155707
12	1	0	0.375272	1.593020	-1.836958
13	1	0	-3.383714	1.821977	0.267528
14	6	0	1.211613	2.520271	0.711270
15	1	0	1.401017	2.077450	1.694400
16	1	0	2.030038	2.268449	0.040209
17	1	0	1.185203	3.604478	0.864167
18	6	0	-1.105675	-0.838234	1.560853
19	7	0	-1.069674	-0.805954	2.718704
20	6	0	1.417456	-0.479464	-0.389354
21	6	0	1.936376	-0.660403	0.892929
22	6	0	2.316134	-0.393204	-1.457327
23	6	0	3.312507	-0.724596	1.100164
24	1	0	1.283672	-0.736310	1.752329
25	6	0	3.687996	-0.451456	-1.253460
26	1	0	1.935866	-0.265270	-2.467133
27	6	0	4.194088	-0.608214	0.033336
28	1	0	3.691851	-0.857888	2.107820
29	1	0	4.361116	-0.374679	-2.100729
30	1	0	5.265211	-0.648002	0.199999
31	7	0	-2.194860	-1.487795	-0.412050
32	8	0	-2.275736	-1.590125	-1.652701
33	8	0	-3.093998	-1.933829	0.326329

TS2B

Center Number	Atomic Number	Atomic Type	Coordinates (Angstroms) X	Y	Z
1	6	0	-0.464178	2.046631	0.180143
2	6	0	-0.454581	1.219957	-1.035917
3	6	0	-2.634141	1.716953	-0.319752
4	6	0	-1.779277	2.242845	0.614450
5	1	0	-2.063033	2.720086	1.541959
6	6	0	-1.909229	1.238789	-1.510443
7	1	0	-2.286310	0.291452	-1.900952
8	1	0	-2.034224	1.979986	-2.311404
9	6	0	-0.043447	-0.311870	-0.696489
10	1	0	-0.189459	-0.804724	-1.658034
11	6	0	-1.017309	-0.959530	0.219314
12	1	0	0.274182	1.577549	-1.764457

13	1	0	-3.713391	1.692936	-0.225940
14	6	0	0.735873	2.574172	0.853282
15	1	0	1.652066	2.091038	0.520187
16	1	0	0.807902	3.642063	0.609692
17	1	0	0.638075	2.505201	1.938699
18	6	0	-1.104928	-0.687111	1.590977
19	7	0	-1.116336	-0.392972	2.712973
20	6	0	1.438586	-0.405605	-0.364934
21	6	0	1.936577	-0.778422	0.881719
22	6	0	2.351856	-0.100451	-1.378999
23	6	0	3.308656	-0.820634	1.114370
24	1	0	1.265441	-1.039170	1.689628
25	6	0	3.719801	-0.141665	-1.150096
26	1	0	1.988915	0.168233	-2.367270
27	6	0	4.204968	-0.496966	0.104887
28	1	0	3.673578	-1.110678	2.093917
29	1	0	4.407432	0.097768	-1.954085
30	1	0	5.273249	-0.528999	0.289573
31	7	0	-1.954156	-1.841638	-0.283430
32	8	0	-1.996095	-2.061516	-1.511000
33	8	0	-2.745949	-2.396360	0.495577

PR$_B$

Center Number	Atomic Number	Atomic Type	Coordinates (Angstroms) X	Y	Z
1	6	0	-0.309701	2.239245	-0.219929
2	6	0	-0.423984	1.232983	-1.295667
3	6	0	-2.395823	1.398322	-0.134657
4	6	0	-1.481019	2.238131	0.509022
5	1	0	-1.641890	2.743039	1.451831
6	6	0	-1.940760	1.154523	-1.523160
7	1	0	-2.302229	0.234172	-1.976458
8	1	0	-2.258194	1.996143	-2.150044
9	6	0	-0.064315	-0.247496	-0.726301
10	1	0	-0.228141	-0.875080	-1.601016
11	6	0	-1.089797	-0.685735	0.267291
12	1	0	0.203229	1.419906	-2.166408
13	1	0	-3.379601	1.146024	0.242654
14	6	0	0.886857	3.078143	0.019032
15	1	0	1.806086	2.567720	-0.268586
16	1	0	0.803240	3.967762	-0.617577
17	1	0	0.954756	3.414835	1.054139
18	6	0	-0.950371	-0.545659	1.665394
19	7	0	-0.818270	-0.365655	2.800133
20	6	0	1.399788	-0.379481	-0.351270
21	6	0	1.945438	0.126650	0.829555
22	6	0	2.253312	-1.010473	-1.258719
23	6	0	3.303105	-0.003493	1.098550

24	1	0	1.319229	0.634785	1.553452
25	6	0	3.612798	-1.138377	-0.995269
26	1	0	1.851141	-1.408287	-2.185912
27	6	0	4.142588	-0.636038	0.187757
28	1	0	3.705067	0.396673	2.023345
29	1	0	4.255799	-1.633168	-1.715496
30	1	0	5.202073	-0.735197	0.398687
31	7	0	-2.013156	-1.685113	-0.108259
32	8	0	-2.113667	-2.000676	-1.298764
33	8	0	-2.724208	-2.195272	0.764167
